# Development of Relative Risk Model for Regional Groundwater Risk Assessment: A Case Study in the Lower Liaohe River Plain, China

**DOI:** 10.1371/journal.pone.0128249

**Published:** 2015-05-28

**Authors:** Xianbo Li, Rui Zuo, Yanguo Teng, Jinsheng Wang, Bin Wang

**Affiliations:** 1 College of Water Sciences, Beijing Normal University, Beijing 100875, China; 2 Engineering Research Center of Groundwater Pollution Control and Remediation, Ministry of Education, Beijing 100875, China; NERC Centre for Ecology & Hydrology, UNITED KINGDOM

## Abstract

Increasing pressure on water supply worldwide, especially in arid areas, has resulted in groundwater overexploitation and contamination, and subsequent deterioration of the groundwater quality and threats to public health. Environmental risk assessment of regional groundwater is an important tool for groundwater protection. This study presents a new approach for assessing the environmental risk assessment of regional groundwater. It was carried out with a relative risk model (RRM) coupled with a series of indices, such as a groundwater vulnerability index, which includes receptor analysis, risk source analysis, risk exposure and hazard analysis, risk characterization, and management of groundwater. The risk map is a product of the probability of environmental contamination and impact. The reliability of the RRM was verified using Monte Carlo analysis. This approach was applied to the lower Liaohe River Plain (LLRP), northeastern China, which covers 23604 km^2^. A spatial analysis tool within GIS which was used to interpolate and manipulate the data to develop environmental risk maps of regional groundwater, divided the level of risk from high to low into five ranks (V, IV, III, II, I). The results indicate that areas of relative risk rank (RRR) V cover 2324 km^2^, covering 9.8% of the area; RRR IV covers 3986 km^2^, accounting for 16.9% of the area. It is a new and appropriate method for regional groundwater resource management and land use planning, and is a rapid and effective tool for improving strategic decision making to protect groundwater and reduce environmental risk.

## Introduction

Groundwater is one of the most important sources of drinking water worldwide [1]. However, it has gradually become more and more seriously polluted [2], [3], [4]. Groundwater contamination is a widespread issue associated with the process of urbanization [5], [6], induced degradation of natural groundwater quality and quantity [7], [8]. To evaluate the degree to which groundwater has been affected by human activities, and to provide basic data for groundwater resource management, the concept of a regional environmental risk assessment has been recommended [9], which operates at a spatial scale that contains multiple sources of stressors that affect multiple endpoints [10], [11]. Groundwater environmental risk (GER) assessment (GERA) has been applied in many countries, including India[7], Japan[12], Northern Tunisia[13],] Palestine [14], and so on. In China, it has been applied in Jilin[2], Beijing [5], and other plain areas. The GERA may reflect the regional risk to some degree, but in real application, always generates a qualitative result [15]. Moreover, there are many sources, stressors, receptors, and endpoints at the regional scale that must be considered, and a single stressor often has multiple sources [16], [17]. How to quantify the environmental risk of groundwater has become a research focus of recent years [18].

Usually, a traditional environmental risk assessment, called the four-step method, comprises hazard identification, a dose-response relationship assessment, exposure assessment, and risk characterization, and in the process of evaluation, the stacked index method, process simulation and multivariate statistical methods are commonly used [19]. The data can be managed in Geographic Information Systems (GIS) [20], [21] to facilitate groundwater environmental risk assessment [14].

Based on the risk theory, the relative risk model (RRM) is a powerful tool for risk assessment, is mainly applied to the regional ecological risk assessment (ERA), has been applied in many parts of the world such as Brazil [22], and Australia [23]; it is becoming a recognized and universal method for ERA in China, where it has also been applied in the Haihe River Basin [10], the Hulunbeier [24], and other places. However, it rarely, has been used for groundwater risk assessment. In this study we propose a vulnerability index that is coupled with a RRM that mostly resolves the problem of how to classify the relative risk zones as part of the groundwater environmental risk management process. With defined the study area, receptors, risk sources and exposure, RRM can be used to simulate the distribution of GER values to show the areas that are under pressure. And for groundwater system, RRM has a logical evaluation process, especially with the similar exposure-response relationships [25], the model could be regarded as the basis for regional GERA calculations. By developing the RRM, the distribution of the risk value of the groundwater environment system that is subject to pressure from human activities and land use, and obtained risk zoning maps that can be used for regional planning and development of groundwater resources [26].

The Lower Liaohe River Plain (LLRP), is located in the Northeast Plain, one of China’s four great plains. It is an important industrial area for both Liaoning Province and the whole of the northeast region, and is a designated regional representative site in the Chinese Ecosystem Research Network [27]. However, as the economy has developed rapidly in recent years, issues of water pollution and water quality deterioration have emerged to varying degrees [28], therefore, assessing the groundwater environmental risk in the region is very necessary.

The main purposes of this article are: firstly, to calculate the potential risk from the anthropogenic pollution load, while at the same time, considering the natural vulnerability of the aquifer system; secondly, to conduct a risk assessment of the influence of industrial development and human activities on the regional groundwater system; and thirdly, to complete an environmental risk assessment that is influenced by multi-receptors and multiple sources. The results of this study will be used to optimize the groundwater relative risk assessment model, and will also form the basis for future similar studies.

## Methods

### Study area

**Fig 1:**
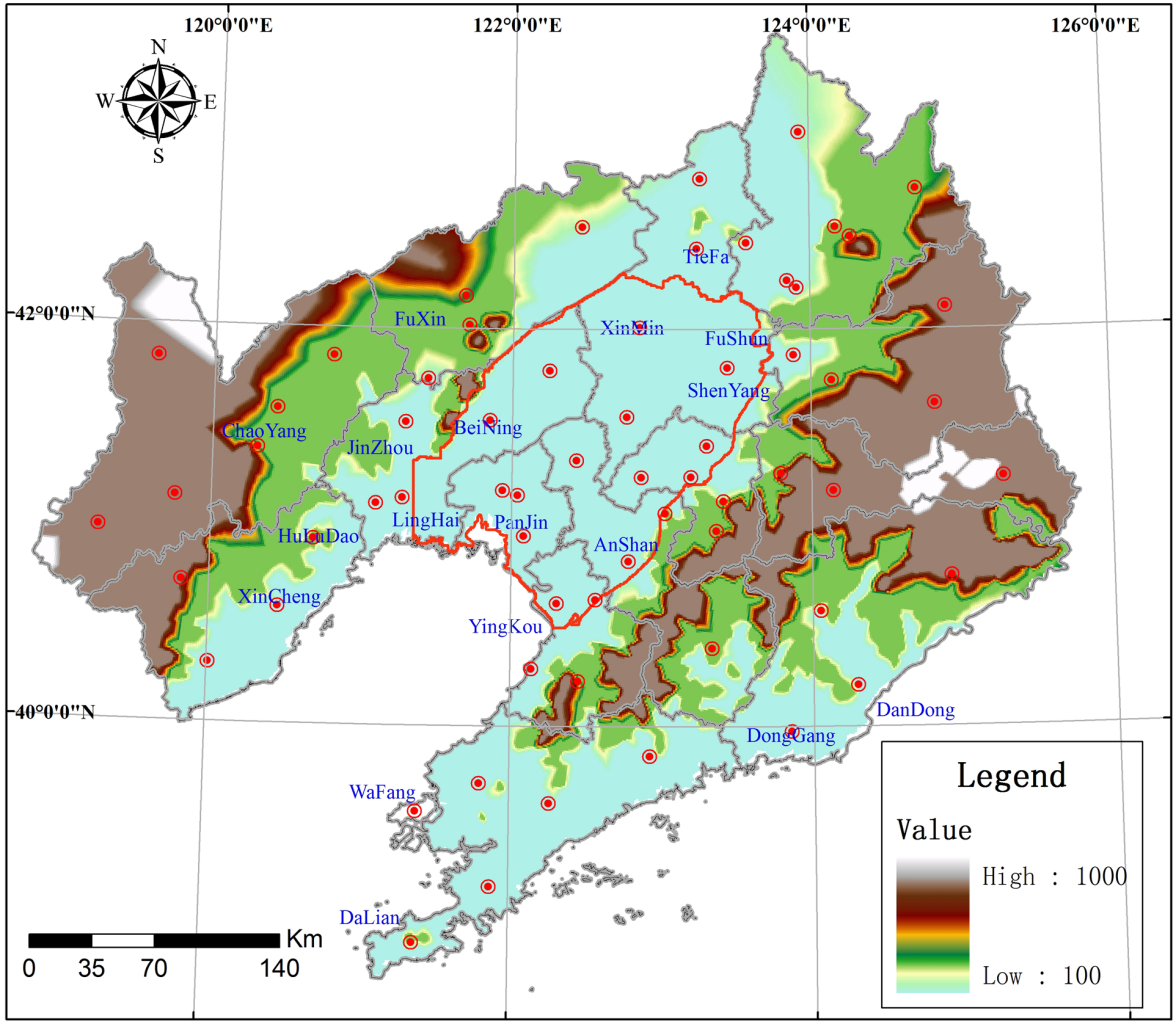
Location of the study area.

The LLRP is located in the central part of Liaoning Province, between 120°00′–123°50′E and 40°30′–42°10′N (Fig 1). It was formed by a river system; detrital material was transported and deposited in the basin as a result of fluvial activity, forming a plain that is surrounded by mountains on three sides, and Liaodong Bay on the southern side. The terrain is generally flat, and elevation decreases from north to south. There are nine prefecture-level cities in the LLRP: Shenyang, Tieling, Fushun, Liaoyang, Anshan, Fuxin, Yingkou, Jinzhou, and Panjin, covering 23604 km^2^. The plain area can be spatially and morphologically divided into the eastern and western Piedmont sloping plains, the central alluvial delta plains, and coastal plains. The Liaohe River Plain is a huge intermontane fault-depression basin. It forms a complete groundwater system with recharge, runoff, and drainage processes. Water systems in the north, east, and west are bounded by exposed bedrock, and by Bohai Bay in the south. The huge vertical thickness can be divided into two subsystems: Quaternary sediments and Cainozoic Bedrock.

### Method development

**Fig 2:**
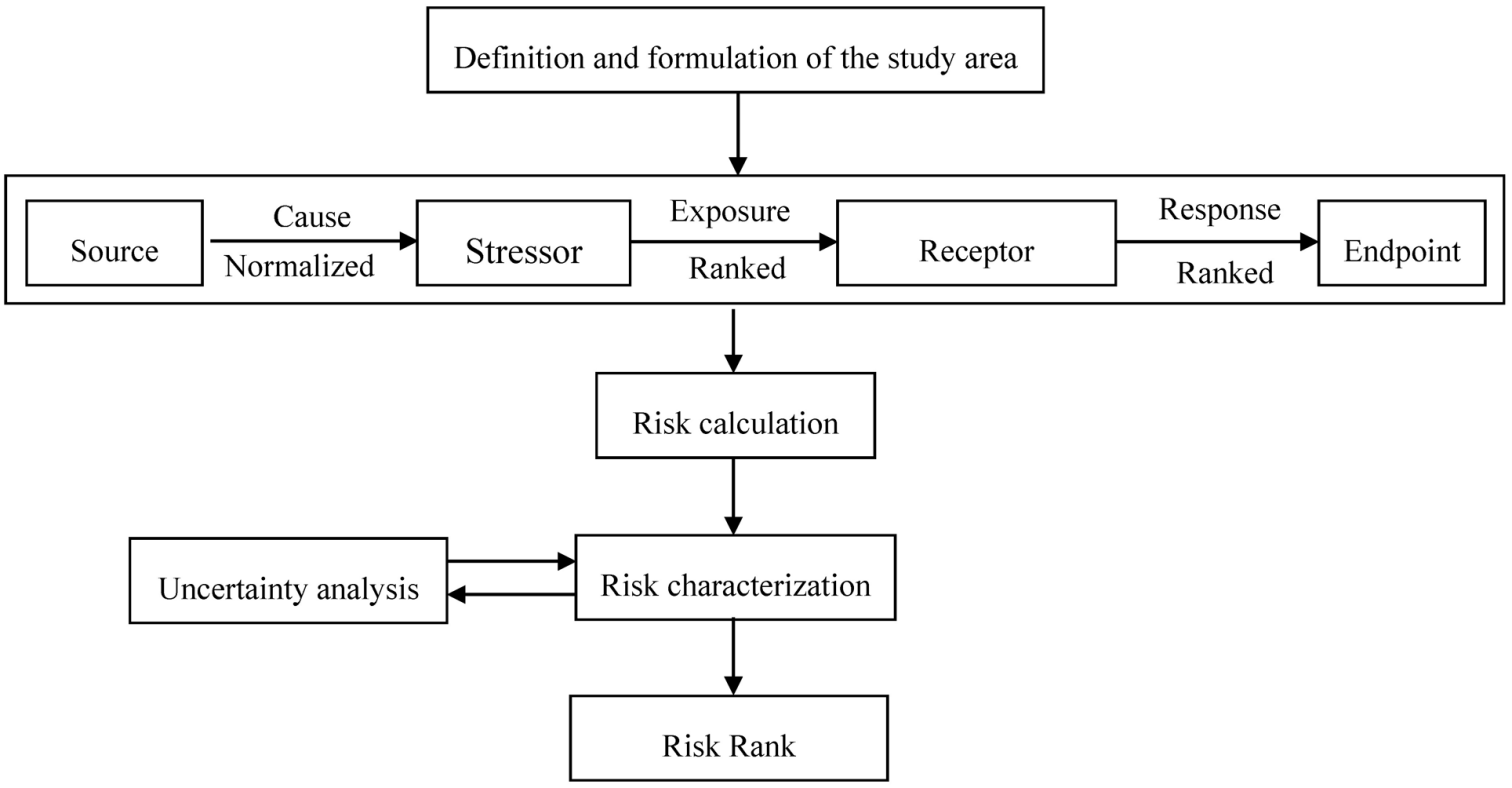
Regional groundwater environment risk assessment procedure.

The procedures for GERA is outlined (Fig 2). With defined groundwater environmental risk problem in the study area, the risk source identification of groundwater was carried on, the groundwater risk receptors should be determined, the exposure and response relationship from the sources to endpoints was analyzed, and the conceptual model of groundwater environmental risk was established. And the risk value could be calculated and divide into different level. Finally, in order to a more reliable result, uncertainty analysis was carried out on the model.

**Fig 3:**
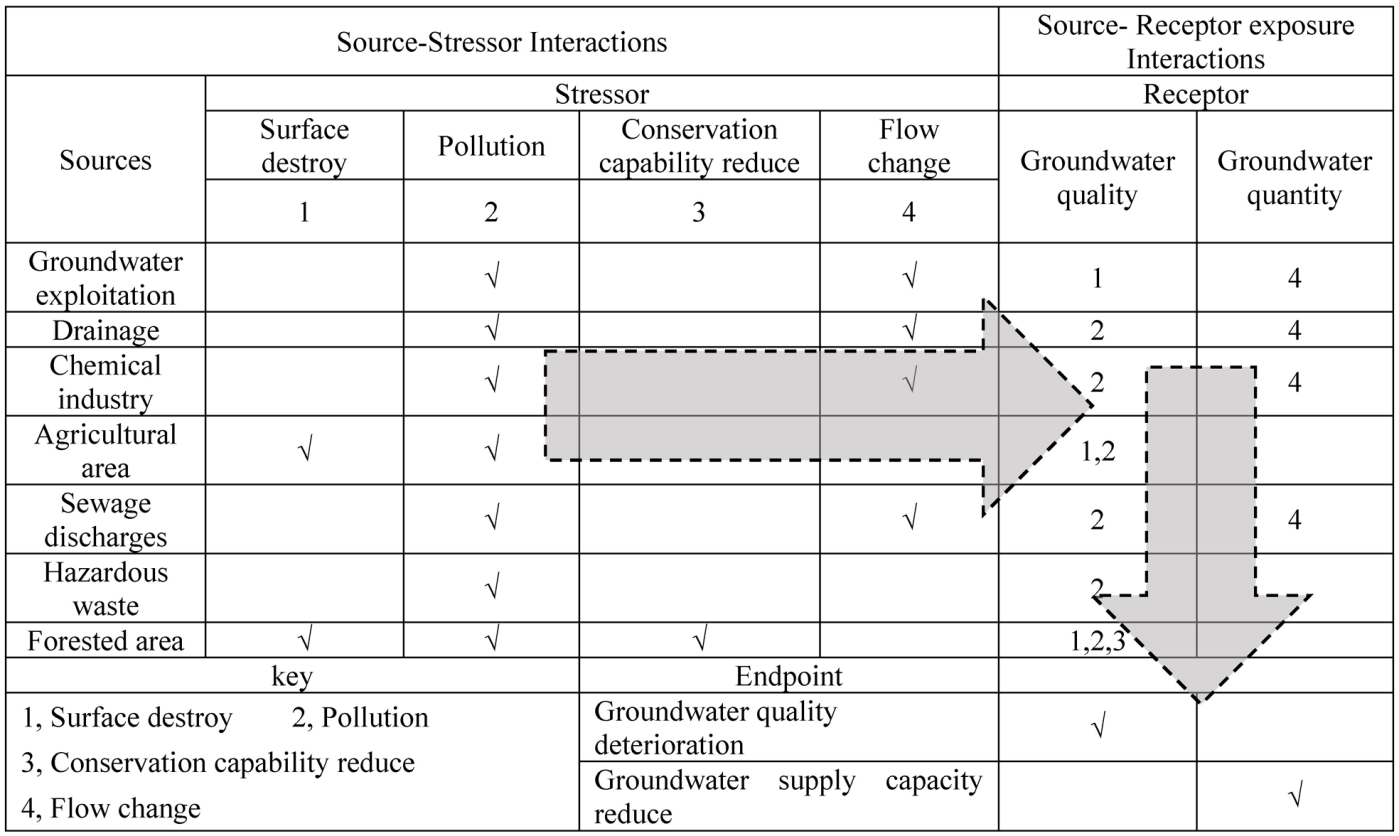
Exposure and response of risk Sources, Receptors, and Endpoints in the regional groundwater conceptual models.

Conceptual model. Regional environmental risk assessment (ERA) is used to evaluate the interaction of three environmental components: sources that release stressors, receptors that bear the risk, and impacts on the endpoints or receptors [29]. Human activities will have a direct or indirect impact on the existing groundwater environment system and ecological processes. The impact is usually manifested as stress on the receptor. Field surveys and data analysis in the study area suggest that the risks to groundwater mainly come from groundwater exploitation, drainage, chemical enterprises, agricultural area, sewage emission, dangerous waste, and forestry, all of these greatly influence the quality and quantity of groundwater via accumulation and pollution, resulting in water quality deterioration and reduction in supply. In this study, the conceptual model of GER was developed by analysis of the groundwater exposure-response pathways (risk source-stressors-Receptor-endpoint) (Fig 3).

Risk source identification. There are many driving factors in risk of the Groundwater. According to research data, we analyze the driving factors from the effects of land use and human activities Based on the field surveys and data research, the principal sources of risk in the study area were divided into seven categories: groundwater exploitation, drainage, chemical enterprises, agricultural area, sewage emission, dangerous waste, and forested area.

(1) Groundwater exploitation

The long-term excessive extraction of groundwater resources has induced the ongoing reduction in groundwater levels in China. The main groundwater funnel area in the study area is relatively large; it covers about 100 km^2^ and extends to Shenyang, Anshan, Liaoyang, and Panjin. In general, the extent of groundwater exploitation is positively corrected with the regional groundwater level, and overdraft leads to the degradation of pasture vegetation, and reduced biodiversity, also eventually leads to seawater incursion and ground subsidence.

(2) Drainage

Because of connectivity between surface water and groundwater, surface water pollution gradually influences groundwater. Rivers in the LLRP include the Liaohe River, Raoyang River, Da Ling River, and the Xiao Ling River. The major river systems in the LLRP receive 8.57 billion t of waste per year[30]. Surface water pollution in this region is already compromising groundwater and coastal water quality, such that groundwater in some wells in Shenyang, Fushun, and Tieling cannot be used because it fails the standards for various contaminants. Part of the groundwater supply along the banks of the Liaohe River comes from easily contaminated shallow groundwater. In addition, the unsaturated zone is subject to undercutting by the river channel.

(3) Chemical enterprises

Industrial pollution of groundwater is a serious and widespread problem in cities in central and eastern China. The main pollution components are the so-called five poisons (volatile phenols, cyanide, arsenic, mercury, and hexavalent chromium) and other heavy metals [30]. By-products from chemical enterprises solid waste dumps, wastewater discharges, and chemical plants can easily enter the groundwater in accidents; indeed, chemical enterprises are positively correlated with the GER. The location of chemical enterprises is the potential risk of groundwater, however, due to the different pollution type and components of each chemical enterprise, in this paper, statistics of the chemical enterprises density in each zone is generalized to risk source.

(4) Agricultural area

The LLRP is an important grain production base in China, however, the actual utilization ratio of chemical fertilizer and pesticide in agricultural production is only 30–40%. And the overuse of chemical fertilizers and pesticides has led to widespread soil pollution, triple-nitrogen, and pesticide pollution of groundwater. Overuse of pesticides and fertilizers influence the groundwater; over-applications of nitrogen and phosphorus fertilizers, to support grain cultivation, have led to excessive nitrogen levels in groundwater in northeastern China.

(5) Sewage emissions

Domestic sewage from urban areas is another major source of groundwater pollution in China. The wastewater emission volume per year in major cities reaches 2.216 billion t in the study area, and accounts for 58.3% of the total provincial volume [30]. Persistent leaks are pervasive throughout sewage systems, leading to pollution of shallow groundwater in urban areas. Meanwhile, the widespread distribution of sewage pits and sinks in small and medium cities lead to nitrogen, chlorine, and organic pollution of groundwater.

(6) Dangerous waste

There are frequent occurrences of serious incidents, such as a lack of measures to control seepage from chromium slag yards, and leakages of arsenic-polluted waste to groundwater, both of which result in serious chromium and arsenic pollution in the regional groundwater. If the larger amount of hazardous waste was produced and stored, coupled with improper disposal, the greater possibility of leachate would percolate into groundwater system.

(7) Forested area

A larger forested area means that there is less anthropogenic disturbance of surface soil, and increased accumulation on the natural forest floor. Forested areas have a greater ability to conserve groundwater, which can effectively prevent surface contaminants from entering the groundwater environment. So, by increasing the area of forested land in the study area, the risk to groundwater can be greatly reduced, which is a key artificial regulation method.

Risk Receptor. Risk Receptor should represent sensitive or important factors in the ecological system. The groundwater value reflects the expected damage to groundwater resources, including water quantity and quality, which compromises the ability to maintain the ecological environment, human health services, and socio-economic services. The groundwater water supply capacity highlights the socio-economic function of the groundwater resource, and the groundwater quality shows the influence of human activities on the groundwater environment. For the groundwater system, any factors, including human activities or natural actions, resulting in the changes, will be reflected in the changes of water quantity and water quality of the groundwater, so in this study groundwater supply capacity and groundwater quality were chosen as the risk receptors, and the decline of groundwater quality and supply were characterized as the risk endpoints.

### Risk analysis

Risk analysis is the process of the mutual interactions among the risk sources, Receptors and risk endpoints.

#### Relative risk source

The relative risk source was indicator representing the frequency and intensity of the sources in different risk region. The value of different sources was calculated as following:

1. Groundwater exploitation intensity was the ratio of the amount of groundwater exploitation and the amount of groundwater allowable withdrawal;
2. Drainage density was the ratio of the length of drainage and the area of risk region;
3. Chemical enterprise density was the ratio of the number of chemical enterprise and the area of different risk region;
4. Agriculture proportion was the ratio of the area of agriculture and the area of risk region;
5. Sewage emission intensity was the ratio of the amount of sewage emission per year and the area of risk region;
6. Dangerous waste intensity was the ratio of the amount of dangerous waste per year and the area of different risk region;
7. Forest proportion was the ratio of the area of forest and the area of different risk region.

And then, each relative risk source was normalized.

#### Receptor ranks

Based on the RRM method, groundwater quality and groundwater supply capacity were selected as risk receptor that have a probability of being under pressure from GER. The receptor ranks was classified using a data segmentation function [10], [31]. In this process, the receptors were ranked into 5 groups, as shown in Table 1.

**Table 1:** The Receptor ranks in the study area.

Criteria ranks	0.111	0.333	0.556	0.778	1
Groundwater quality category	I	II	III	IV	V
Groundwater supply capacity (%)	<25	25–40	40–55	55–70	>70

Note: In “*the Chinese Groundwater Quality Standards*(GB/T 14848–93), which divides the groundwater quality into five categories (I, II, III, IV and V, respectively), indicates the good or bad quality of groundwater, Class I and II is suitable for various purposes, Class III is applicable to centralized drinking groundwater sources, or for industry and agriculture, Class IV and V represents poor water quality, should be strictly processed to use.

#### Exposure analysis of Source-Receptors

Exposure analysis examines the sources of risk in the assessment of regional risk distribution, dynamic and their exposure to the risk Receptors. It is the most important of the regional GER assessment, and it aims to identify the degree of damage from risk sources to groundwater systems and risk Receptors.

As a weighting factor, the source—stressor—receptor exposure filter (SSR) and the stressor-endpoint response filter (SE) is introduced, which are used to determine the relationship between risk components. SSR was acquired based on the following judges: Which stressor will be released by the source? Will the receptor be impacted by the stressor? SE was acquired based on the following judge: How much extent does a particular stressor effect on a certain endpoint? What is the consequence of endpoint? In this study, in order to reflect the meaning of exposure and effect accurately, the exposure filter through four stressors caused by sources was expanded, which were surface destroy, contamination, conservation capacity weak, and flow change. As in similar studies [10], categories of no, lowest, low, medium, high, and highest risk were used to describe SSR and SE on a scale from 0 to 1. A value of 0.9 indicates a clear or validated relationship, 0.7 indicates a relatively strong relationship, 0.5 indicates an obvious relationship, 0.3 indicates less common ground, 0.1 shows that the relationship is not clear, and the value of 0.0 means no relationship at all. SSR and SE are shown in Table 2.

**Table 2:** Exposure and response filters of risk Sources, Receptors, and Endpoints.

Sources	source—stressor—receptor exposure filter (SSR)				Receptors	stressor-endpoint response filter (SE)	
	Surface destroy	Contamination	Conservation capacity weak	Flow change	-	Groundwater quality deterioration	Groundwater supply capacity reduce
Groundwater exploitation	0	0.3	0	0	Groundwater quality	0.3	0
	0	0	0	0.9	Groundwater supply capacity	0	0.7
Drainage	0	0.7	0	0	Groundwater quality	0.7	0
	0	0	0	0.3	Groundwater supply capacity	0	0.5
Chemical industry	0	0.9	0	0	Groundwater quality	0.7	0
	0	0	0	0.3	Groundwater supply capacity	0	0.5
Agricultural area	0.3	0.3	0	0	Groundwater quality	0.5	0
Sewage discharges	0	0.5	0	0	Groundwater quality	0.5	0
	0	0	0	0.3	Groundwater supply capacity	0	0.3
Hazardous waste	0	0.7	0	0	Groundwater quality	0.7	0
Forested area	0.3	0	0.3	0	Groundwater quality	0.5	0

Note: A value of 0.9 indicates a clear or validated relationship, 0.7 indicates a relatively strong relationship, 0.5 indicates an obvious relationship, 0.3 indicates less common ground, 0.1 shows that the relationship is not clear, and the value of 0.0 means no relationship at all.

At the same time, with considering the regional difference that stressor acted on receptor and each receptor accepted the stressor, the SSR of each risk region was different. And the exposure extant difference of the same type source among the different regions was adjusted based on the groundwater vulnerability index (GVI).

The GVI describes the resistance to groundwater pollution, and it has been commonly assessed by the DRASTIC evaluation index [32], [33], [34]. Seven hydrological geological indicators, depth of groundwater (D), aquifer net recharge (R), aquifer lithology (A), soil type (S), topography (T), impact of the vadose zone (I), and aquifer hydraulic conductivity coefficient (C), were selected to quantitatively analyze groundwater vulnerability in the model; they were chosen because of their high potential to influence aquifer vulnerability. Each segment was allocated a number from 1 to 10, depending on its importance, and each index was allocated a weight depending on its influence on vulnerability, and the final vulnerability index was the composite of the seven indicators (Eq 1), known as P.

$$\text{P}\;={\;\text{D}}_{\text{W}}{\text{D}}_{\text{V}}+{\;\text{R}}_{\text{W}}{\text{R}}_{\text{V}}+{\;\text{A}}_{\text{W}}{\text{A}}_{\text{V}}+{\;\text{S}}_{\text{W}}{\text{S}}_{\text{V}}+{\;\text{T}}_{\text{W}}{\text{T}}_{\text{V}}+{\;\text{I}}_{\text{W}}{\text{I}}_{\text{V}}+{\;\text{C}}_{\text{W}}{\text{C}}_{\text{V}}$$ (1)

where P = groundwater vulnerability index(GVI), subscripts V and W indicate the corresponding ratings and weights. DI, the relative GVI is:

$$\text{DI}\;=\;\text{P}/{\text{P}}_{\text{max}}.$$ (2)

The model assumes that contaminants from the surface penetrate into the groundwater; pollutants infiltrate into the groundwater with rainfall; contaminants flow with water, and the assessment area is bigger than 40 km^2^.

### Risk characterization

#### Risk calculation

When the RRM model is used to evaluate the GER, sources of risk in the region and Receptors should be included in the pressure assessment and analysis. In addition, interpretation should be based on the following assumptions:

1. The sensitivity of risk Receptors to stressors varies with Receptor type, and the Receptor sensitivity is positively correlated with the response to stress;
2. There is a positive correlation between frequency of the risk source and the release pressure; a higher density of Receptors linked to the endpoint will result in a greater likelihood of exposure to pressure.
3. Effects on groundwater risk endpoint and effect in the same area of the multiple risk pressure can be superimposed according to its relative level of risk.

In this process, the sensitive exposure factor and the response factors was analyzed firstly, and this analysis was followed by cumulative analysis and regional GER division. The extent of the risk to groundwater depends on the source of the contaminated groundwater and the resistance of the aquifer to pollution.

The RRM model was used to evaluate the regional GER by accumulating integrated calculations of the relative risk source value, SSR filters, the DI, the receptor rank, and the SE filters. The risk value was calculated using the following formula(Eq 3) in ArcGIS software, and the section assignment method was used for risk ranking in the study area.

$$R_{v}=\;\sum^{\text{​}}\;\left(S_{i}\times SSR_{in}\times DI\times R_{m}\;\times SE_{ml}\right)$$ (3)

$$RRv=Rv/Rv_{max}$$ (4)

Here, *Rv* is the risk value; *RRv* is the relative risk value; *i* is the source series; *n* is the stressors series; *m* represents the receptor series; *l* represents the endpoint series; *R*_*m*_ is the receptor rank in sub-regions; *S*_*i*_ is the relative risk source value in sub-regions; *DI* is the relative GVI; *SSR*_*in*_ the source—stressor—receptor exposure filter in sub-regions; *SE*_*ml*_ is the stressor-endpoint response filter in sub-regions.

#### Risk characterization

Risk characterization is the final stage in the process of GRA that provides overall final risk scores for each sub-region, source, Receptor, and endpoint through integrating exposure and effect data [10]. This process summarizes the analyses of the study area, Receptor, risk source and risk exposure, and the relationships between them. Meanwhile, the results of every analysis stage are combined in this process.

The Relative Risk Value (RRV) are normalized by dividing by the maximum value. The groundwater risk was divided into five ranks from Relative Risk Rank (RRR) I to V according to the RRV [0–0.2),[0.2–0.4),[0.4–0.6), [0.6–0.8), [0.8–1], these different ranks correspond to lowest, low, medium, high, and highest risk levels.

### Uncertainty analysis

The uncertainty of the groundwater system itself introduces uncertainties to the methods for dividing the region, choosing the Receptors, identifying the main sources of risk, selecting the assessment endpoints and the evaluation methods. The entire GER assessment process and results are associated with uncertainty, so uncertainty analysis of the GER assessment is a necessary step. In this study, there are two important considered uncertainties, one is the uncertainty associated with assigning quantitative values to various sources of risk because of limited data, while the other is the impact of various risk sources on risk levels.

The uncertainty was addressed using Monte Carlo analysis. Monte Carlo is a prediction model that measures the distribution of the model input parameters using an uncertainty indicator and produces a variable probability distribution curve of the uncertainty. When the lateral span of the probability distribution is large, the confidence interval of the uncertainty model is small, or vice versa. Monte Carlo uncertainty analysis is frequently used in RRM models [10], [28].

Monte Carlo simulations of the uncertain model parameters were run for 1000 iterations using Crystal Ball 2000. Output distributions were derived for input sources, Receptors, and endpoints. The distributions show a range of probable risk associated with each point estimate.

## Results and Discussion

### Sample analysis and quality control

(1) Background data

In this study, data of agricultural and forest land, the sewage and dangerous waste discharges in the LLRP were obtained from “Liaoning Province Statistical Yearbook 2013”. Values for groundwater exploitation and the river network density were obtained from the China Groundwater Resources Data System and Liaoning Province Water Resources Bulletin 2012. The risk density of chemical enterprises in the district was calculated from the Inventory of Chemical Plants in Liaoning Province 2012.

(2) Sampling and analysis

**Fig 4:**
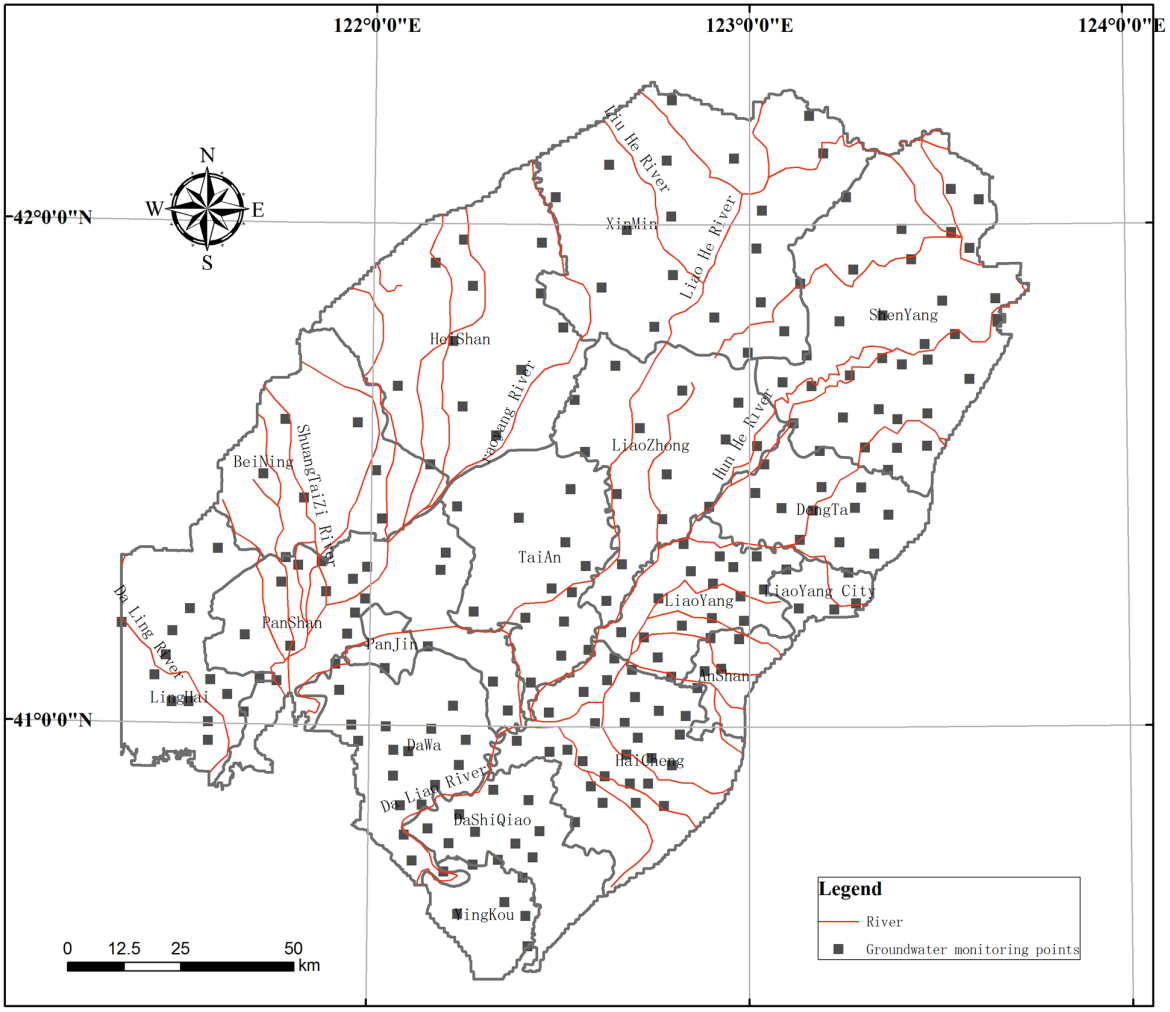
Location of groundwater monitoring points.

In this study, the data of hydrogeological drilling and shallow groundwater quality analysis results are done in collaboration with Geological Survey Institute of Liaoning Province. There was a total of 228 groundwater monitoring point in the region (Fig 4), including 99 new shallow water drilling wells, 129 self-contained water source wells. And the locations of new boreholes, with careful layout scheme, are permitted by Land and Resources Bureau of Liaoning Province. All of the drilling and sampling work according to China’s laws and regulations, was not related to the natural reserve and special, endangered or protected species. In the process of field investigation, 228 shallow groundwater level value was collected, and 228 groundwater samples were analyzed.

### Calculation results

#### Relative risk source

**Fig 5:**
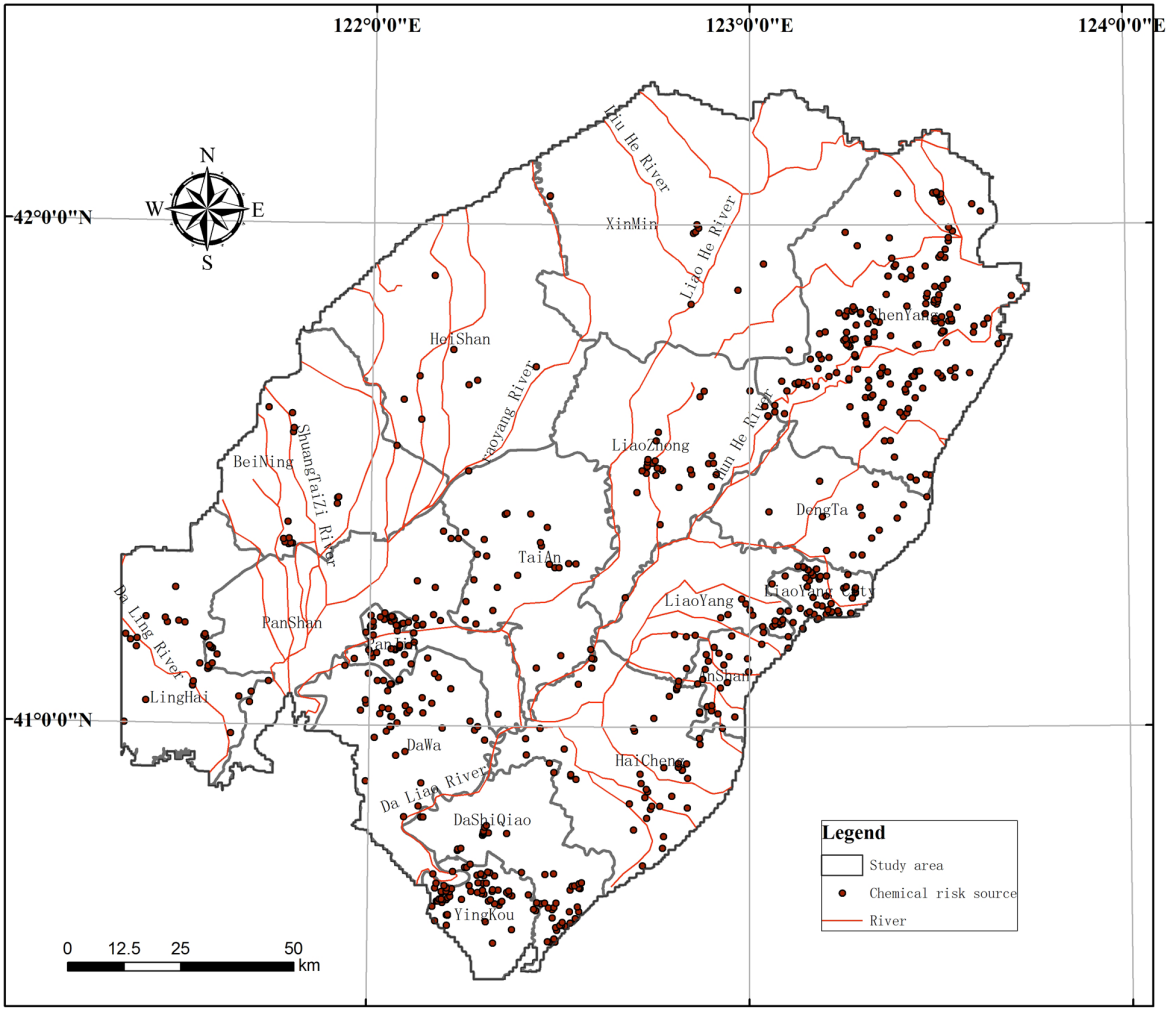
Distribution of the chemical enterprises in the study area.

**Fig 6:**
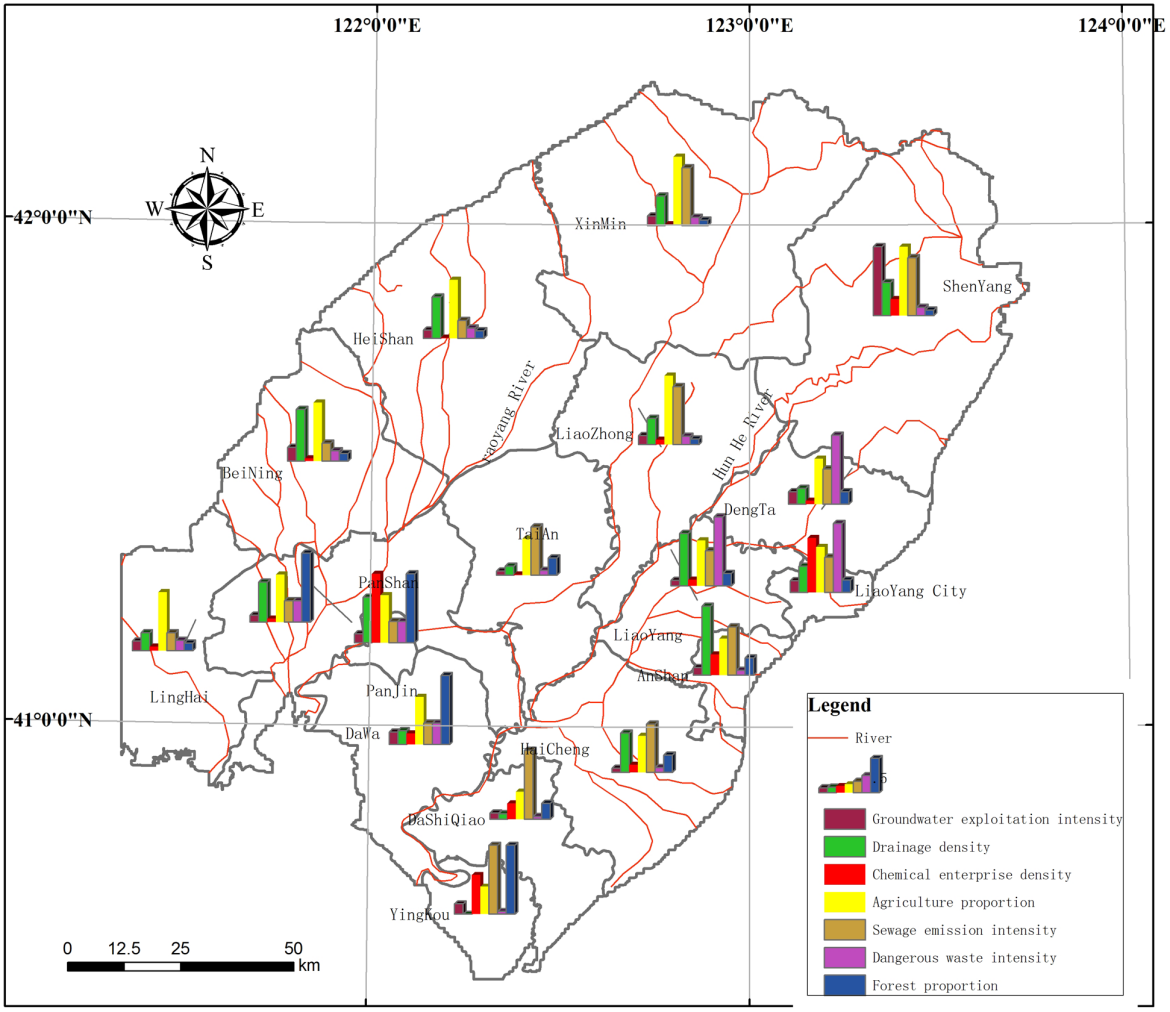
Risk source normalization in the study area.

Eighteen risk regions were identified based on the administrative counties of the LLRP. The risk source status is shown in Table 3 below. According to the planning of groundwater exploitation, each city or county, there is a requirement of mining limit, in the LLRP, the extent of groundwater exploitation ranged from 27.96% to 473.53%, and, with the exception of Shenyang, groundwater was not overexploited. The maximum river network density value was 0.128 km/km^2^, in Jinzhou. The maximum chemical enterprise density was 0.26 a/km^2^, in Jinzhou and Panjin (Table 3) and (Fig 5), and the maximum agricultural area density was 5.15%, in the central part of Liaoning Province and Xinmin. Meanwhile, the maximum sewage discharge was in Yingkou, and amounted to 52,200 t/km^2^/yr, and the maximum volume of dangerous waste was produced in Liaoyang, and was 83.08 t/km^2^. The maximum forested area was in Shengyang, accounting for 2.24%. The risk values for the sources after normalization are shown (Fig 6).

**Table 3:** Sources of risk in the Lower Liao River.

No	Risk region	Groundwater exploitation intensity (%)	Drainage density (km/km^2^)	Chemical enterprise density (per/km^2^)	Agriculture proportion (%)	Sewage emission intensity (10^4^t/km^2^)	Dangerous waste intensity (t/km^2^)	Forest proportion (%)
1	XinMing	64.78	0.084	0.004	5.18	4.37	9.56	2.24
2	LiaoZhong	60.93	0.074	0.018	5.18	4.37	9.56	2.24
3	ShenYang	473.53	0.094	0.063	5.18	4.37	9.56	2.24
4	HeiShan	54.52	0.117	0.003	4.42	1.34	12.22	1.53
5	BeiNing	93.9	0.146	0.010	4.42	1.34	12.22	1.53
6	LingHai	65.62	0.051	0.017	4.42	1.34	12.22	1.53
7	JinZhou	36.04	0.066	0.260	4.42	1.34	12.22	1.53
8	PanJin	60.89	0.128	0.260	3.56	1.62	25.38	0.17
9	PanShan	46.34	0.113	0.014	3.56	1.62	25.38	0.17
10	DaWa	86.21	0.040	0.041	3.56	1.62	25.38	0.17
11	YingKou	70.82	0.000	0.148	2.05	5.22	2.93	0.17
12	DaShiQiao	42.79	0.016	0.060	2.05	5.22	2.93	0.74
13	HaiYun	27.96	0.110	0.027	2.75	3.66	5.79	0.68
14	TaiAn	28.19	0.024	0.002	2.75	3.66	5.79	0.68
15	AnShan	49.78	0.194	0.079	2.75	3.66	5.79	0.68
16	DengTa	85.33	0.044	0.014	3.44	2.63	83.08	0.95
17	LiaoYang	39.24	0.147	0.023	3.44	2.63	83.08	0.95
18	LiaoYangCity	81.2	0.073	0.204	3.44	2.63	83.08	0.95

#### Groundwater vulnerability index

**Fig 7:**
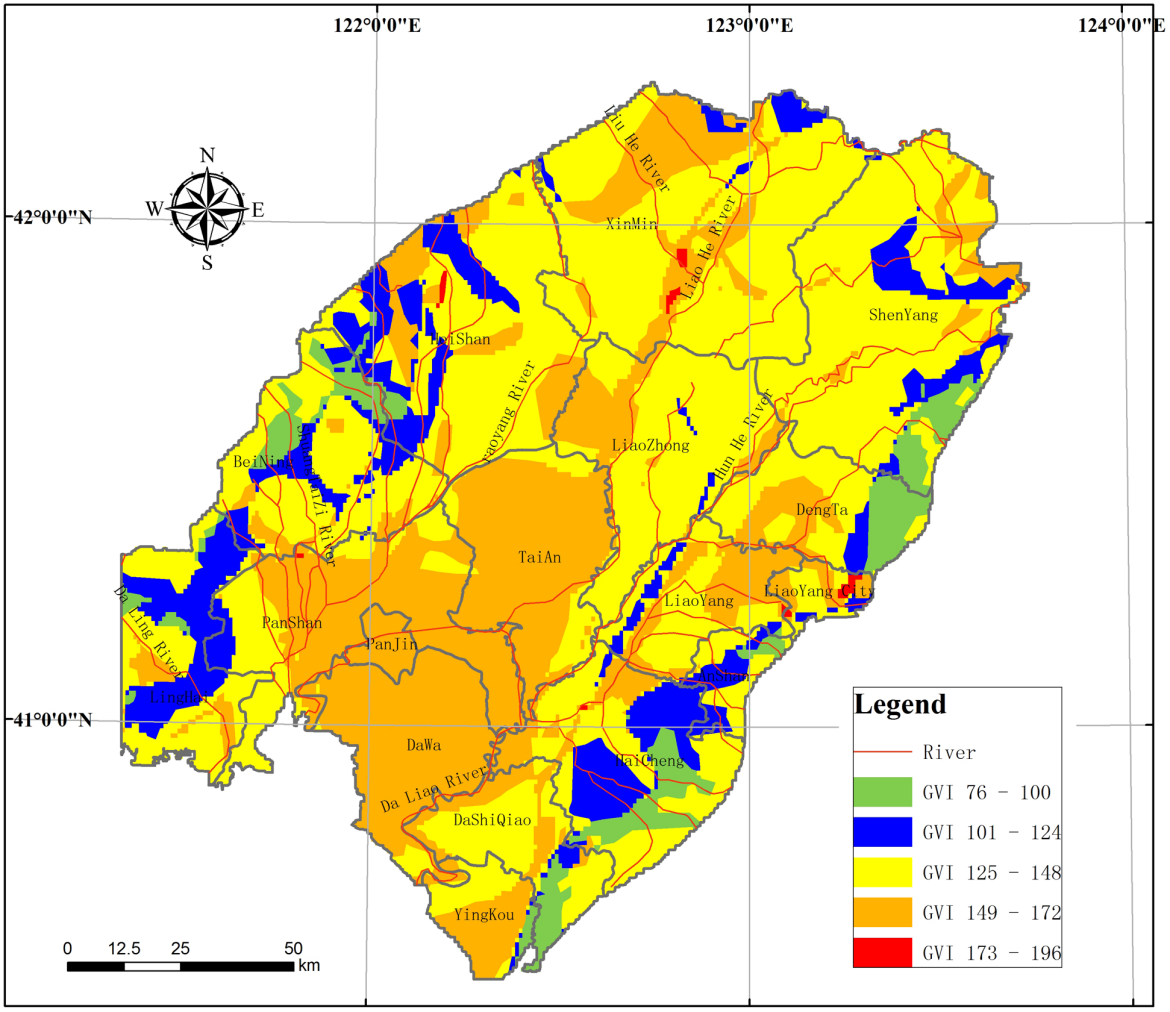
Groundwater vulnerability index(GVI) in the study area.

The GVI in the LLRP was calculated as follows: firstly, the seven parameters that determined the GVI were identified and the relevant data were collected, and secondly, the parameters were ranked according to certain standards to obtain figures for the different compartments; then the DRASTIC index calculation formula was used to calculate the distribution of the DRASTIC index for the seven compartments of the study region, and finally, the DRASTIC index was classified so that maps could be produced using certain standards. The GVI of the compartments in the study area is showed (Fig 7).

**Fig 8:**
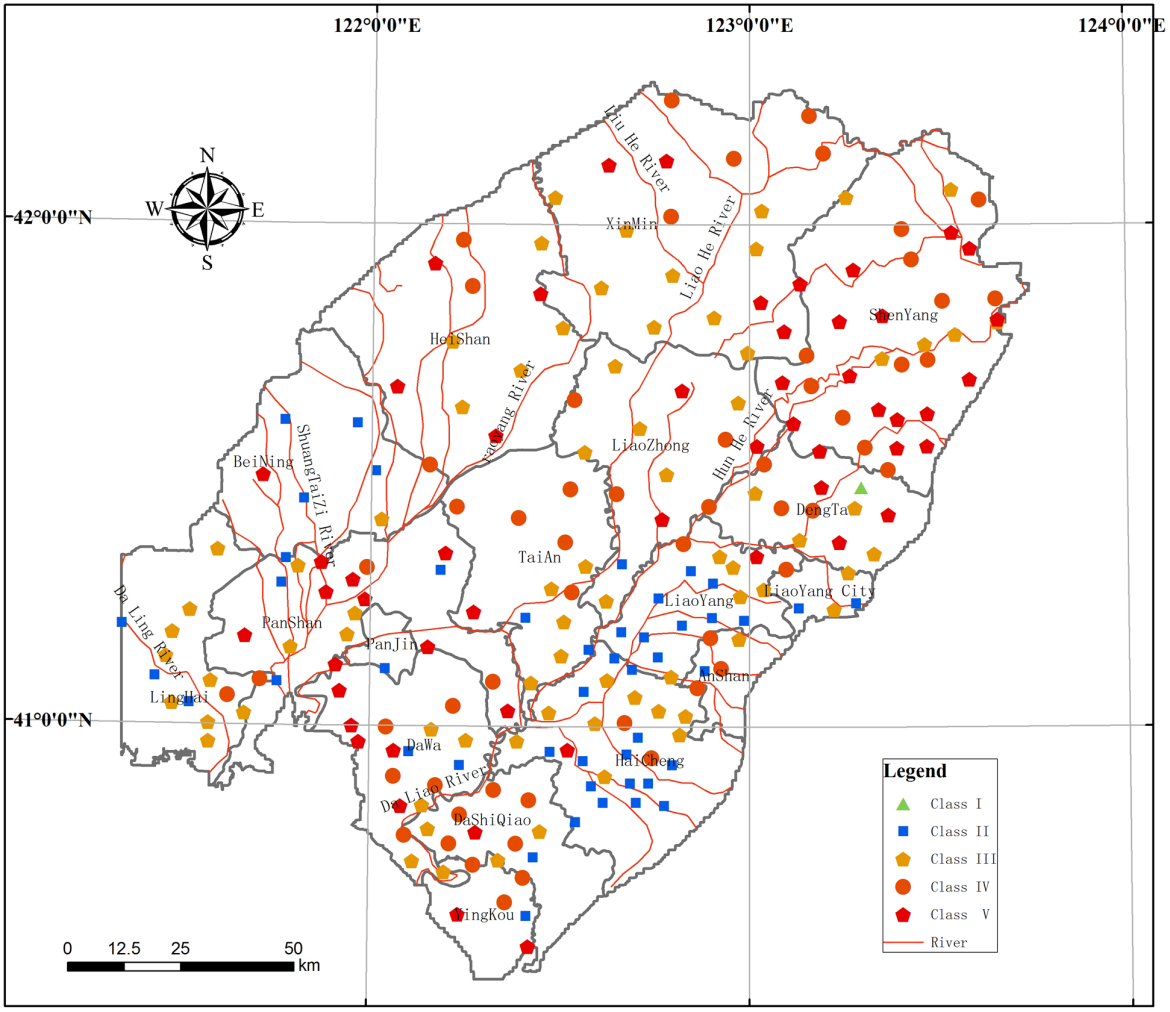
Comprehensive evaluation of groundwater quality in the study area.

Risk Receptor. Shallow groundwater in the LLRP quality was evaluated by comparing 228 groundwater samples in *“the Chinese Groundwater Quality Standards(GB/T 14848–93)*[35], which divides the groundwater quality into five categories (I, II, III, IV and V, respectively), indicates the good or bad quality of groundwater, Class I and II is suitable for various purposes, Class III is applicable to centralized drinking groundwater sources, or for industry and agriculture, Class IV and V represents poor water quality, should be strictly processed to use. Overall, the groundwater quality is CLASS V in Shenyang, CLASS IV in Panjin and Yingkou, and CLASS III in the west and the southeast of the Liao River Plain. In the different groundwater system compartments, the quality is CLASS II in the eastern and western Piedmont sloping plains, CLASS III in the central alluvial plain, CLASS IV in the Ling River fan and in the central coastal plain of the Southern Bin Sea Plain, and CLASS V in the Liao River alluvial fan and the Hun River alluvial fan (Fig 8) The most frequently reported pollutants are iron, manganese, and ammonia-nitrogen. The main sources of groundwater pollution are sewage, industrial waste-water effluent, municipal solid waste, agricultural pollutants, and mining activities. Groundwater accounts for 70.78% of the water supply in Shenyang, and 89.2% of the water supply in Jinzhou (Table 4).

**Table 4:** The groundwater supply status of the study area.

Administrative district	Surface water supply percent (%)	Groundwater supply (%)			
	-	Water volume for life	Water volume for agriculture	Water volume for industry	Total (%)
ShenYang	29.22	4.13	10.51	3.5	70.78
AnShan	61.39	0.25	1.31	0.78	38.61
FuShun	91.06	0.1	0.3	0.31	8.94
JinZhou	10.8	0.87	5.82	1.4	89.2
YingKou	87	0.12	0.68	0.11	13
LiaoYang	48.34	0.73	4.64	1.48	51.66
PanJin	90.38	0.02	0.96	0.11	9.62

Note: Groundwater supply accounted for the percentage of the total water supply

#### Map of relative risk assessment

**Fig 9:**
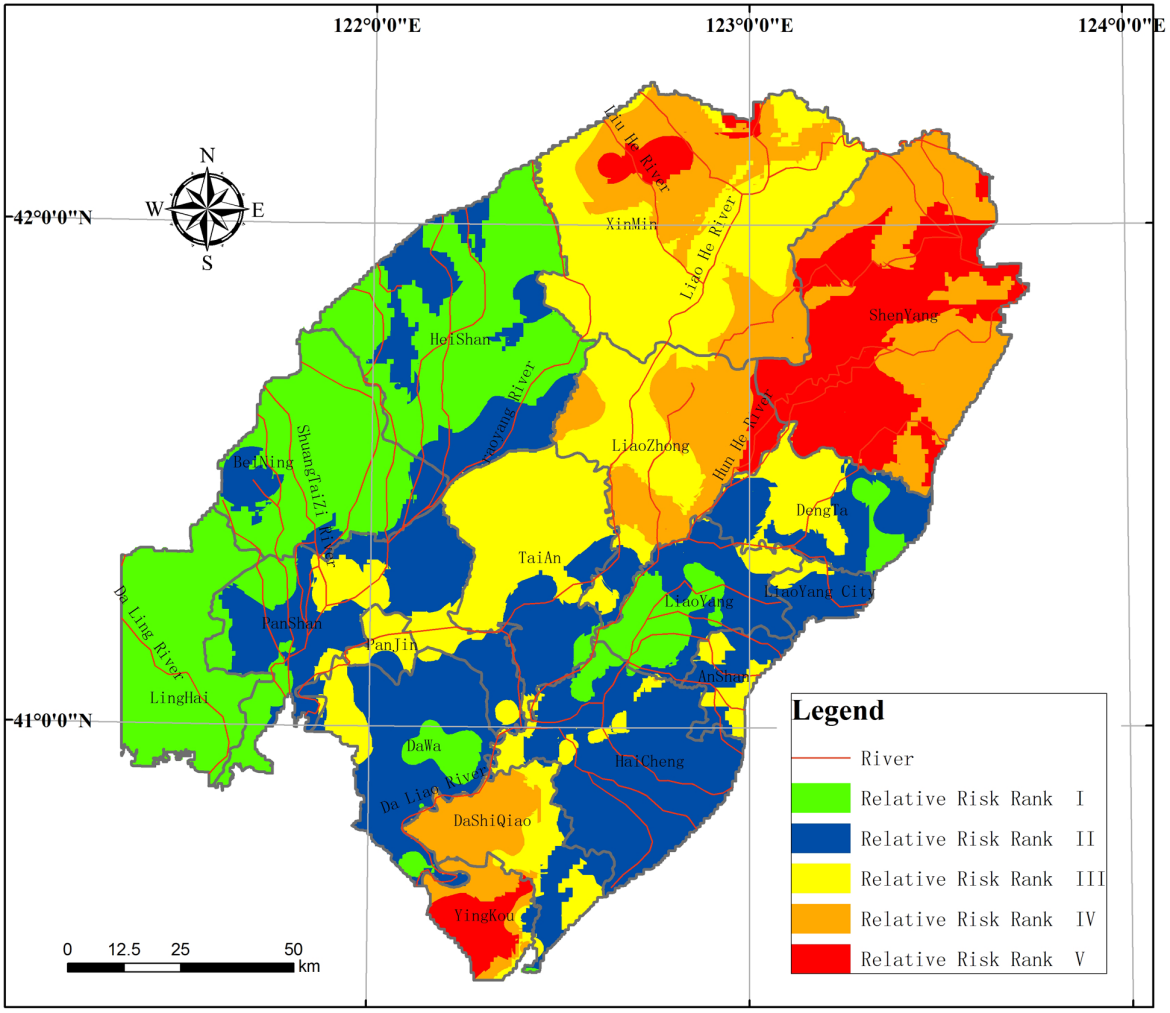
Regional Groundwater Environment Risk assessment RRR in the study area.

The results of the relative risk assessment for the LLRP are shown (Fig 9) and Table 5. The RRR V areas are mainly in the Hun River alluvial-proluvial fan in Shenyang; it covers 2324km^2^, with 9.8% of the region, with the reason of high supply of groundwater resources in the area, and the groundwater resources therefore need to be protected in this area. Because of the low (some parts are medium) values for the vulnerability index, industrial solid waste and waste from well-developed regional chemical enterprises is not effectively used or disposed of, so groundwater is polluted to different degrees. Further, some of the groundwater engineering facilities and systems for checking water measures are not complete, which means that there is aquifer pollution from surface sewage, and different aquifers are polluted by the same contaminants.

**Table 5:** Regional Groundwater Environment Risk assessment RRR in the study area.

No	Relative Risk value(RRV)	relative risk rank(RRR)	Risk level	Area (km^2^)	Proportion (%)
1	[0–0.2)	I	lowest	6028	25.5
2	[0.2–0.4)	II	low	5762	24.4
3	[0.4–0.6)	III	medium	5504	23.3
4	[0.6–0.8)	IV	high	3986	16.9
5	[0.8–1]	V	highest	2324	9.8

The RRR IV areas are mainly located in Liaoning Province, in Xinmin and Yingkou, and cover 3986 km^2^, accounting for 16.9% of the area. In this area the degree of risk is relative higher. The groundwater is widely contaminated by fertilizers and pesticides through soil percolation, moreover, long-term applications of sewage for irrigation are compromising farmland and groundwater quality. The situation is exacerbated by excessive use of ammonia nitrogen, nitrite nitrogen and nitrate nitrogen, and there is organic pollution in agricultural areas.

The RRR III areas are distributed in the central plains, on both sides of the Liao River, and the RRR II areas are found in the southeastern coastal areas, meanwhile the RRR I area is in the western mountains, where there is relatively low risk to groundwater environment.

### Uncertainty risk analysis

**Fig 10:**
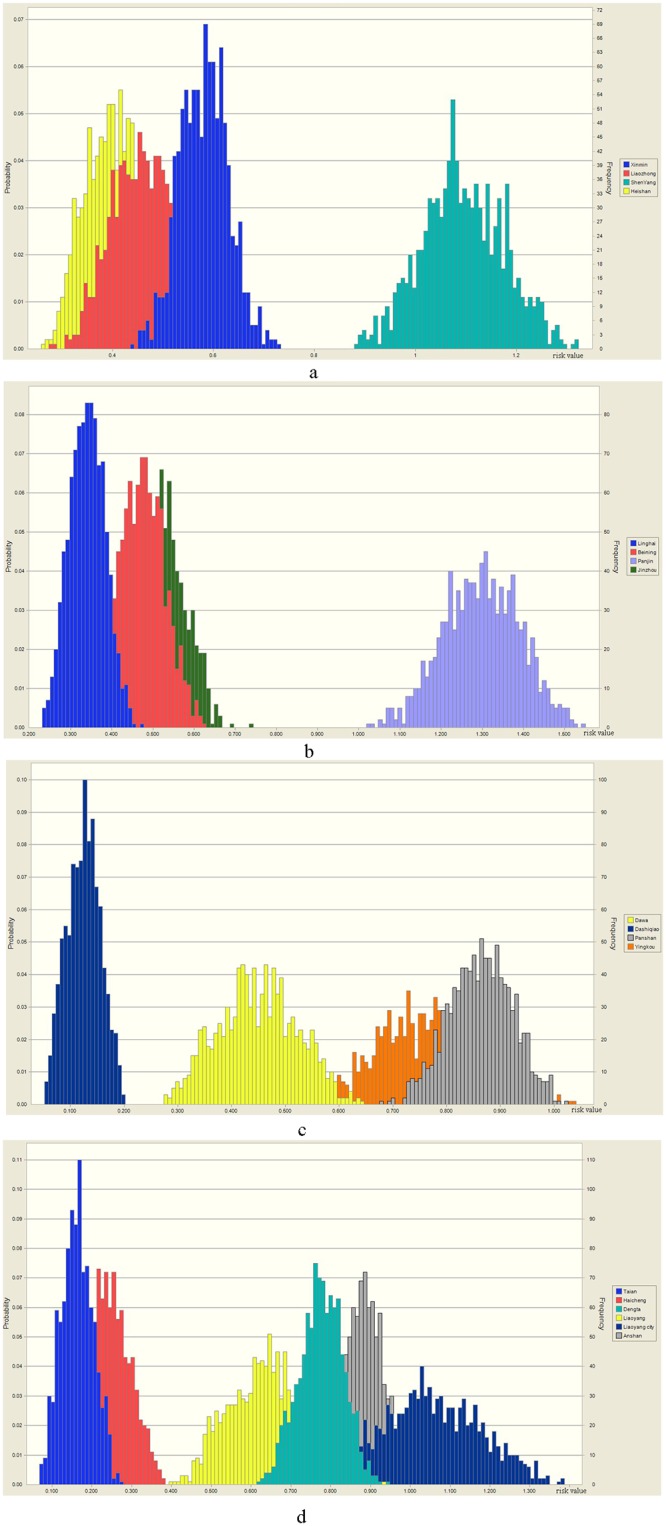
Uncertainty of probability distribution of Monte Carlo relative risk value calculations.

The relative risk value of 18 selected communities is shown in Table 6. Results show that the risk source and DI values vary by about 10%. The probability is normally distributed, and the risk receptors-groundwater quality and groundwater exploitation volume are likely to vary within a level according to the field survey values. Using Crystal Ball 2000 software macros in Excel 2003 software, with 1000 Monte Carlo iterations, the output uncertainties of the probability distribution were plotted (Fig 10).

**Table 6:** The relative risk value calculations for representative points.

No	Position of representative points	GEI	DD	CED	AP	SEI	DWI	FP	DI	GQC	GS	RV	RRV	RRR
1	XinMing	0.14	0.43	0.02	1.00	0.84	0.12	0.08	0.71	0.778	0.778	0.580	0.41	III
2	LiaoZhong	0.13	0.38	0.07	1.00	0.84	0.12	0.08	0.74	0.556	0.778	0.475	0.34	II
3	ShenYang	1.00	0.48	0.24	1.00	0.84	0.12	0.08	0.68	1.000	0.778	1.161	0.82	V
4	HeiShan	0.12	0.60	0.01	0.85	0.26	0.15	0.11	0.68	0.556	1.000	0.405	0.29	II
5	BeiNing	0.20	0.75	0.04	0.85	0.26	0.15	0.11	0.67	0.556	1.000	0.487	0.34	II
6	LingHai	0.14	0.26	0.06	0.85	0.26	0.15	0.11	0.70	0.556	1.000	0.349	0.25	II
7	JinZhou	0.14	0.34	1.00	0.85	0.26	0.15	0.11	0.52	0.556	1.000	0.520	0.37	II
8	PanJin	0.10	0.66	1.00	0.69	0.31	0.31	1.00	0.80	1.000	0.111	1.381	0.98	V
9	PanShan	0.10	0.58	0.05	0.69	0.31	0.31	1.00	0.85	1.000	0.111	0.918	0.65	IV
10	DaWa	0.18	0.20	0.16	0.69	0.31	0.31	1.00	0.80	0.556	0.111	0.438	0.31	II
11	YingKou	0.18	0.00	0.57	0.40	1.00	0.04	1.00	0.88	0.778	0.111	0.752	0.53	IV
12	DaShiQiao	0.09	0.08	0.23	0.40	1.00	0.04	0.23	0.50	0.333	0.111	0.119	0.08	I
13	HaiYun	0.06	0.57	0.11	0.53	0.70	0.07	0.25	0.73	0.333	0.333	0.242	0.17	I
14	TaiAn	0.06	0.13	0.01	0.53	0.70	0.07	0.25	0.78	0.333	0.333	0.165	0.12	I
15	AnShan	0.06	1.00	0.30	0.53	0.70	0.07	0.25	0.76	1.000	0.333	0.936	0.66	IV
16	DengTa	0.18	0.23	0.05	0.66	0.51	1.00	0.18	0.73	1.000	0.556	0.833	0.59	III
17	LiaoYang	0.08	0.76	0.09	0.66	0.51	1.00	0.18	0.75	0.556	0.556	0.431	0.30	II
18	LiaoYang City	0.08	0.38	0.79	0.66	0.51	1.00	0.18	0.78	0.778	0.556	0.628	0.44	III

GEI, Groundwater exploitation intensity; DD,Drainage density. CED,Chemical enterprise density; AP, Agriculture proportion; SEI, Sewage emission intensity; DWI, Dangerous waste intensity; FP, Forest proportion; DI, the relative groundwater vulnerability index; GQC, Groundwater quality category; GS, Groundwater supply; RV, risk value; RRV, Relative risk value; RRR, Relative risk rank.

Monte Carlo simulations show that, among the seven separate risk source effects, the lateral span of the probability distribution is between 0.15 and 0.54. The range of the relative values is the greatest in Liaoyang City (0.82–1.36) and the probability is the largest when the risk value of 1.08, while the range is smallest for Dashiqiao. In general, the uncertainty is normally distributed, and the risk prediction values fluctuate between the averages in each region. These fluctuations do not influence the conclusions or change the RRM prediction results.

**Fig 11:**
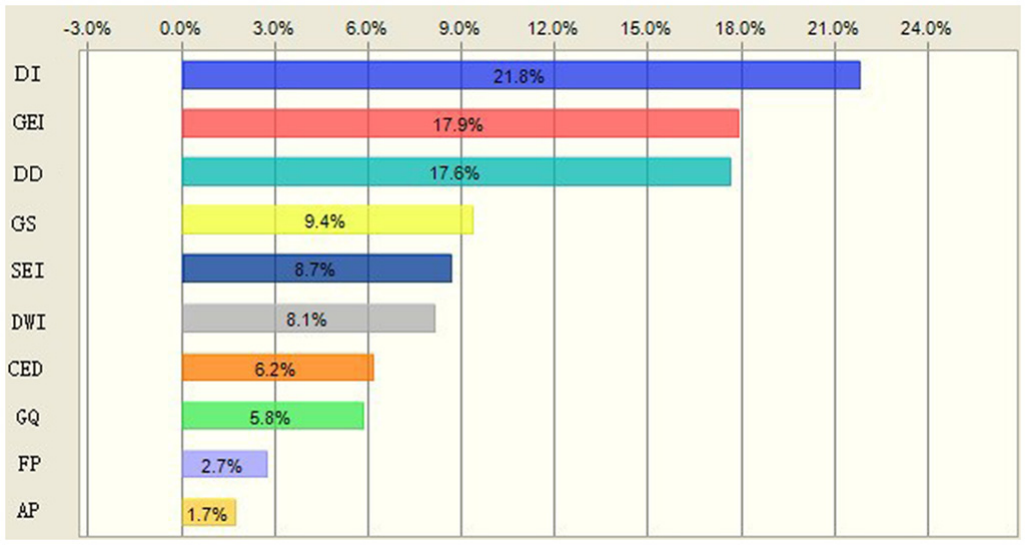
Sensitivity analyses on factors of relative risk models for representative points in Xinmin. DI, Relative groundwater vulnerability index; GEI, Groundwater exploitation intensity; DD, Drainage density; GS, groundwater supply; SEI, Sewage emission intensity; DWI, Dangerous waste intensity; CED, Chemical enterprise density; GQ, groundwater quality; FP, Forest proportion; AP, Agriculture proportion.

Xinmin has a larger relative risk value, so we carry out further analysis of its risk sources and receptors, and sensitivity analysis of GVI. Monte Carlo simulation results show that the sources of uncertainty are widely distributed (Fig 11). The GVI, groundwater exploitation intensity, drainage density, groundwater supply capacity, and the sewage discharge contributions accounted for 21.8%, 17.9%, 17.6%, 9.4%, and 8.7% of the uncertainty, respectively. The results of ranking the uncertainty contributions associated with the relative risk values for Xinmin are shown (Fig 11).

### Conclusions

Environmental risk assessment of regional groundwater is an important tool for groundwater protection, which can be applied at different scales, and which, when coupled with groundwater vulnerability index, should be complete, relevant, and functional. In this study, a RRM of regional GERA, which is similar to ecological risk assessment procedures, including the definition and formulation of the study area, receptor analysis, risk source analysis, exposure and hazard analysis, risk characteristics, and risk management, was developed by analyzing the exposure response pathways of the regional groundwater environment. The risk sources were groundwater exploitation, drainage, chemical enterprises, agricultural area, sewage emission, dangerous waste, and forestry. And the risk receptors were groundwater quality and groundwater supply capacity, the risk endpoints were deterioration in groundwater quality and reduced groundwater supply. The RRM accumulated the relative risks in the assessed region, through integrated calculations of the relative risk source value, SSR filters, the DI, the receptor rank, and the SE filters.

As a case study, the RRM of the GER assessment was used to demonstrate the present state of the groundwater environment in the LLRP. By developing the RRM model and simulating the pressures of human activities and land use, the distribution of groundwater environment risk values were obtained. The area of RRR with level V is 2324 km^2^, with 9.8% of the region, and is mainly located in the Hun River alluvial-proluvial fan in Shenyang, Central Liaoning Province. Xinmin and Yingkou are classified as RRR IV; this category covers 3986 km^2^, with 16.9% of the study region. Areas of RRR III are distributed in the central plains, scattered along both sides of the Liao River. And the southeastern coastal area is classified as RRR II, while the western mountains are classified as RRR I, where is relatively low risk to groundwater environment.

Uncertainty analysis using Monte Carlo simulations showed that, among the seven separate effects from the risk sources, the lateral span of the probability distribution is between 0.15 and 0.54. The range of the risk values is greatest in Liaoyang City (0.82–1.36), while it is smallest in Dashiqiao. In general, the uncertainty is normally distributed; the risk prediction scores fluctuate between the averages for each region, and the fluctuation does not affect the conclusions or change the RRM prediction results.

In conclusion, RRM is an approximate analysis of the ranks and distribution of the risk of multiple sub-regions, sources, receptors and endpoints; and the mapped results provide sufficient spatial information at the regional scale. It is a new and appropriate method for regional groundwater resource management and land use planning, and is a rapid and effective tool for improving strategic decision making to protect groundwater and reduce environmental risk.
